# Effect of fermentation and postcooking procedure on quality parameters and volatile compounds of beef jerky

**DOI:** 10.1002/fsn3.1515

**Published:** 2020-04-08

**Authors:** Yulong Luo, Lihua Zhao, Junqiang Xu, Lin Su, Zhimin Jin, Rina Su, Ye Jin

**Affiliations:** ^1^ College of Food Science and Engineering Inner Mongolia Agricultural University Huhhot China; ^2^ The Inner Mongolia Food and Drug Administration Food Inspection Test Center Huhhot China

**Keywords:** fermentation, jerky, postcooking procedure, volatile compounds

## Abstract

In this study, jerky samples were prepared from seasoned beef with and without the addition of starter culture. They were then dried or fried and analyzed to identify the quality parameters and volatile flavor compounds. Samples (starter and control) were divided into drying (inoculated‐drying, I‐D; noninoculated‐drying, N‐D) and frying (inoculated‐frying, I‐F; noninoculated‐frying, N‐F) groups. Water activity (*a*_w_), lightness (L*), and redness (a*) values were significantly affected (*p* < .001) by the postcooking procedures (drying or frying). Hardness, chewiness, and resilience were significantly lower in the dried groups than in the fried groups (*p* < .05). The formation of volatile compounds was affected by cooking treatment, with the highest levels observed in the I‐F group. After frying, the most abundant flavor compounds in the I‐D and N‐D groups were terpenes and sulfur‐containing compounds, followed by aromatic hydrocarbons, ketones, and alcohols. The most common compounds among all groups were acetoin, D‐limonene, anethole, styrene, and tetramethylpyrazine. Overall, the jerky in the I‐F group had the best color and tenderness scores among all groups.

## Graphical abstract

The influence of cooking procedure and fermentation on quality characteristics and volatile compounds of beef jerky were studied. The representative substance in beef jerky was confirmed. The present starter can improve beef jerky quality and flavor.

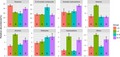

## 1 INTRODUCTION

Beef jerky is among the most popular meat products consumed by humans. Manufactured by salting and drying, it is distinguished by its flavor and texture and is a convenient and healthy snack food (Choi et al., 2008). Beef jerky has become popular and is readily found in retail shops worldwide, occupying a considerable market share (Banout, Kucerova, & Marek, 2012).

Dried meat snack foods have favorable sensory attributes and an appropriate shelf life. The most important sensory attributes of beef jerky are its texture, color, and flavor (Konieczny, Stangierski, & Kijowski, 2007). More than 1,000 volatile compounds develop in jerky upon heating (Pegg & Shahidi, 2004), and these compounds are derived mainly from lipid oxidation and Maillard reactions. Maillard reactions are dramatically accelerated at temperatures above 140°C, due to dehydration of the meat surface (MacLeod & Seyyedainardebili, 1981). However, only volatile compounds such as pyrazines, imidazoles, thiophenes, and furans play significant roles in overall flavor development, due to their very low odor thresholds and characteristic aroma.

Jerky has traditionally been prepared from chopped muscle that is salted and dried without starter cultures. Current methods include the addition of microbial starter cultures at the salting and ripening stage (Scheinberg, Svoboda, & Cutter, 2014). After fermentation, jerky had the unique taste and flavor, and fishy smell had been solved effectively (Jia, Ma, Liu, & Kong, 2014). Starter cultures (e.g., *Lactobacilli* and *Micrococci*) are actively involved in the development of texture, color, and flavor (Fadda, López, & Vignolo, 2010; Lorenzo, Gómez, & Fonseca, 2014). These cultures can contribute to improved safety and flavor quality (López, Sentandreu, Vignolo, Vignoloa, & Fadda, 2015; Lorenzo, Gómez, Purriños, & Fonseca, 2016) and significantly reduce fermentation time. Bacterial proteases and peptidases contribute to the initial breakdown of myofibrillar proteins, resulting in the release of small peptides and amino acids, which have beneficial effects on health and flavor (Bermúdez, Franco, Carballo, Sentandreu, & Lorenzo, 2014; Jin et al., 2015; Mora et al., 2015). Those small peptides and amino acids, including aspartic (Asp), serine (Ser), Glu‐Asp, and Glu‐Ser, contribute directly to umami.

The postcooking procedure (drying or frying) is a critical step in beef jerky processing because it reduces water activity, thereby extending shelf life (Choi et al., 2008). Postcooking procedures also have a major influence on tenderness and flavor, due to the denaturation of myofibrillar components, and promote structural changes and collagen solubilization (Christensen, Purslow, & Larsen, 2000; Walsh et al., 2010). Drying is a traditional jerky postcooking procedure in China. However, frying beef jerky can produce unique flavors, including mellow, caramel, and roasted note (Tornberg, 2005).

The objective of this study was to investigate the quality parameters and formation of volatile compounds in beef jerky and their dependency on the postcooking procedure and commercial starter culture.

## 2 MATERIALS AND METHODS

### 2.1 Preparation of meat and curing solution

The semitendinosus beef (Inner Mongolia native steer, 24 months old) was purchased from a local processor. Visible subcutaneous fatty and connective tissue was removed using a sterile knife, and approximately 5 × 2 × 2 cm strips were prepared. The beef samples contained the following additives (g/kg): soy sauce (5), salt (5), sugar (5), glucose (1), black pepper (5), ginger powder (5), onion powder (5), monosodium glutamate (1), and sodium nitrate (0.002). A commercial dried starter culture composed of *Pediococcus pentosaceus*, *Pediococcus acidilactici*, *Staphylococcus carnosus*, and *Staphylococcus xylosus* was provided by Clerici Sacco Group (SBW‐52, Clerici Sacco).

### 2.2 Production of beef jerky

The main jerky manufacturing processes applied in this study included fermentation and the postcooking procedure (Table 1), performed according to the method of Liu et al. (2016). Seasoned beef was mixed with starter culture, tumbled for approximately 15 min (5 g culture/1 kg beef), and then sealed with plastic wrap and placed in an incubator at 4°C for 24 hr of salting. The samples were then fermented at 25°C and 95%–98% relative humidity for 24 hr, after which the temperature and relative humidity were lowered to 15°C and 85%–95%, respectively, for 48 hr. Finally, the postcooking procedures (drying or frying) were performed, respectively. In the drying process, the device used was a drier, generating hot air of 100°C. The drying time of the product under these conditions was 1 hr. In the drying process, meat strips were fried 0.5 hr at 100°C.

**Table 1:** The processing methods of jerky

	Temperature (°C)	Time (h)	Relative humidity (%)
Fermentation	25	24	95–98
Ripening	15	48	95–85
Frying	100	0.5	—
Drying	100	1	—

Note

Postcooking procedure includes drying and frying, and these groups were divided into drying and frying groups.

Four meat treatments (500 g each) were prepared. Two groups were inoculated with starter culture (5 g culture/1 kg beef), and two groups were used as controls. These groups were further divided into four drying and frying subgroups: inoculated‐frying (I‐F), inoculated‐drying (I‐D), noninoculated‐frying (N‐F), and noninoculated‐drying (N‐D).

### 2.3 Measurements of color, pH, and water activity

The color of the beef jerky samples was analyzed using a colorimeter (TP2 Minolta Chroma Meter, Konica Minolta Sensing Inc.) calibrated with a standard white plate (D65 light source; *Y* = 92.6, *x* = 0.3162, *y* = 0.3324) overwrapped with the applicable film. Four colorimeter measurements were obtained on each sample. The color values were measured at different locations on the sample from each group, including lightness (L*), redness (a*), and yellowness (b*); All measurement locations were taken on the skin side surface in an area free of obvious color defects.

The pH of the jerky was determined using a pH meter (PB‐10, Sartorius) after blending a 5 g sample with 20 ml distilled water for 60 s in a homogenizer (Ultra‐Turrax T25, Akribis Scientific Ltd), as described by Lee et al. (2012) with some modifications. Measurements were conducted in triplicate for each group.

Sample *a*_w_ values were determined in triplicate using a hygrometer at 25°C (LabMaster‐aW, Novasina).

### 2.4 Texture profile analysis (TPA)

Texture profile analysis was conducted following the method of Omana, Moayedi, Xu, and Betti (2010) using the TA.XT Express Stable Micro Systems Texture Analyser (Stable Microsystems Ltd.) equipped with the Texture Expert program. Five cubes (~1 cm^3^) were prepared from every group. A double compression cycle test was performed to 40% compression of the original portion height using an aluminum cylinder probe (36 mm diameter). A time of 5 s was allowed to elapse between compression cycles. Force–time deformation curves were obtained for a 30‐kg load cell applied at a crosshead speed of 2 mm/s. The following parameters were quantified: hardness maximum force required to compress the sample, springiness, ability of the sample to recover its original form after deforming force was removed, cohesiveness, extent to which the sample could be deformed prior rupture, chewiness, energy required to chew a solid for swallowing, and resilience, be defined as how well a product regain its original position.

### 2.5 Analysis of volatile compounds

Solid‐phase microextraction (SPME) and gas chromatography/mass spectrometry (GC/MS) were used to analyze volatile compounds, as described by Marušić, Vidaček, Tibor, Petrak, and Medić (2014). Jerky samples (5 g) were ground using a grinder and homogenized in a blender in 5 ml distilled water saturated with NaCl. This mixture was placed into 15‐ml vials tightly capped with a polytetrafluoroethylene septum. A magnetic stirrer was added to the mixture for stirring during extraction.

The SPME fiber was coated with a 50/30‐μm layer of divinylbenzene/carboxen/polydimethylsiloxane (Supelco), heated in the injection port at 250°C for 30 min, and checked for interference or carry over before use. Volatiles were extracted from 5‐g ground jerky samples in 15‐ml glass sample vials at 50°C for 45 min. After extraction, the fiber was withdrawn into the needle and immediately injected into the GC/MS system and desorbed for 4 min at 250°C. Volatiles were analyzed in triplicate. Analyses were performed using the Trace 1300 Series GC fitted with the ISQ MS on the Xcalibur Chemstation (Thermo Fisher Scientific). The injection port, held at 250°C, was used to thermally desorb volatiles from the SPME fiber onto the front of the capillary column (30 m × 0.25 mm; film thickness: 0.25 μm) (TR‐5MS; Thermo Fisher Scientific). Helium was used as the carrier gas at a flow rate of 1.0 ml/min. The injector was used in the splitless mode. The temperature was set at 40°C and maintained for 3 min, increased to 150°C at 4°C/min and maintained for 1 min, increased to 200°C at 5°C/min, increased from 200 to 250°C at 10°C/min, and the final temperature was held for 5 min. The transfer line temperature and ion source temperature were maintained at 250°C. Mass spectra were obtained at 70 eV and a scan range of 30–400 m/z. Volatile compounds were identified by comparison with mass spectrum data from the National Institute of Standards and Technology library database (MS Search 2.0). The quantities of the volatile compounds were approximated by a comparison of their peak areas with that of the 2‐methyl‐3‐heptanone internal standard, which was obtained from total ion chromatograms using a response factor of 1.

### 2.6 Statistical analysis

Four samples were randomly selected from each group, and each sample was tested in triplicate. Data were analyzed using the general linear model (GLM) procedure (SPSS 19.0) with fixed effects of fermentation, cooking procedures, and fermentation × cooking procedures interaction in a two‐way analysis of variance. Statistical analyses were performed using SPSS 19.0 software (SPSS Inc.). All results are expressed as means ± *SE*.

## 3 RESULTS AND DISCUSSION

### 3.1 Quality characteristics in beef jerky

The food safety of jerky depends on the drying process of the seasoned meat and on the appropriate selection of the spice blend; these processes ensure an appropriate *a*_w_ value (<0.85) (Zhao et al., 2011). The beef jerky *a*_w_ value typically decreases throughout the ripening process due to dehydration. In this study, the range of the jerky *a*_w_ values were 0.83–0.86 (Table 2), at which most microorganisms cannot grow; thus, the growth of hazardous bacteria was inhibited. There was no significant difference in *a*_w_ values between the inoculated and noninoculated groups (*p* > .05). The *a*_w_ values of the fried jerky samples were lower than those of the dried jerky samples (*p* < .001) and did not exceed 0.85 (Table 2), due to postcooking dehydration. High‐temperature frying conditions caused rapid denaturation of the myofibrillar proteins accumulated in the beef jerky, resulting in rapid evaporation of water. Juárez et al. (2010) observed that different cooking methods resulted in moisture loss, which was most effective with frying followed by drying.

**Table 2:** Quality characteristics in beef jerky prepared with different fermentation (F) and cooking procedures (C) (*n* = 4)

	I‐F	N‐F	I‐D	N‐D	*SEM*	F	C	F × C
Aw	0.835^a^	0.835^a^	0.855^b^	0.856^b^	0.003	NS	***	NS
pH	5.56^a^	5.60^a^	5.55^a^	5.58^a^	0.011	NS	NS	NS
L*	20.19^a^	19.99^a^	22.24^b^	22.17^b^	0.322	NS	***	NS
a*	16.45^d^	15.23^b^	16.20^c^	14.76^a^	0.209	***	***	*
b*	6.42^a^	6.45^a^	6.35^a^	6.34^a^	0.022	NS	NS	NS
Hardness (N)	20.334^ab^	22.328^b^	17.444^a^	18.651^ab^	0.775	NS	*	NS
Springiness	0.784^a^	0.941^b^	0.849^ab^	0.881^b^	0.021	*	NS	NS
Cohesiveness	0.710^a^	0.768^a^	0.659^a^	0.674^a^	0.021	NS	NS	NS
Chewiness (N)	11.424^a^	16.129^b^	9.949^a^	11.044^a^	0.896	NS	*	NS
Resilience	0.373^ab^	0.425^b^	0.328^a^	0.318^a^	0.016	NS	*	NS

Note

^a–d^Means within the same row with different superscript upper case letters are different (*p* < .05).

Abbreviations: nd: not detected; ns, not significant; *SEM*, standard error of the mean.

**p < .01.

* *p* < .05.

*** *p* < .001

Color, an important trait in beef jerky, was affected by cooking and fermentation (Table 2). Our jerky color values were consistent with those reported by Konieczny et al. (2007). Fried jerky had higher L* values (*p* < .001) and lower a* values than those of dried jerky (*p* < .001), indicating that frying results in a darker meat color than drying, which was related to the degree of Maillard reaction during frying (Barbut, 2013). The addition of a bacterial starter culture resulted in an increase in surface color values, especially a*. The a* values were higher in the IF group than in all other groups (*p* < .001). Starter cultures with nitrate reductase activity were able to reduce nitrite to nitric oxide, which then reacted with myoglobin‐forming nitrosomyoglobin, a pigment characteristic of cured meats (Bosse, Gibis, Schmidt, & Weiss, 2016; Ruiz, Villanueva, Favarotrindade, & Contreras‐Castillo, 2014).

Low pH values can inhibit or delay microbial spoilage of various dried meat products. The pH values of the jerky samples ranged from 5.55 to 5.60 and did not differ significantly (*p* > .05) among the groups, which are consistent with the findings of Unklesbay, Unklesbay, and Clarke (1999) for beef snack sticks. It is likely that the effect of the starter culture was limited to the jerky surface, because the incubation period during tumbling was brief.

The textural properties of the beef jerky samples are shown in Table 2. Jerky hardness values ranged from approximately 17.444 to 22.328 N, indicating that the variation in textural properties was affected by moisture content (Herrero et al., 2007; Lorenzo, Montes, Purriños, & Franco, 2012). Springiness ranged from 0.784 to 0.941. Resilience values showed less variation, ranging from 0.318 to 0.425. Fried jerky had higher hardness, chewiness, and resilience values, but higher springiness values, than those of dried jerky (*p* < .05). The formation of texture is mainly the result of acid‐induced gelation of muscle proteins, and the amount of water released (Zeng, Xia, Jiang, & Yan, 2013). Tenderness is associated with TPA parameters, and the tenderness was higher in the inoculated than noninoculated groups (Table 2). In the presence of starter culture, jerky pH generally decreases, allowing solubilized myofibrillar proteins to aggregate to form a gel during fermentation. This process leads to the formation of an ordered protein network, which in turn contributes to firmness (González‐Fernández, Santos, Rovira, & Jaime, 2006).

### 3.2 Analysis of volatile compounds

**Figure 1:**
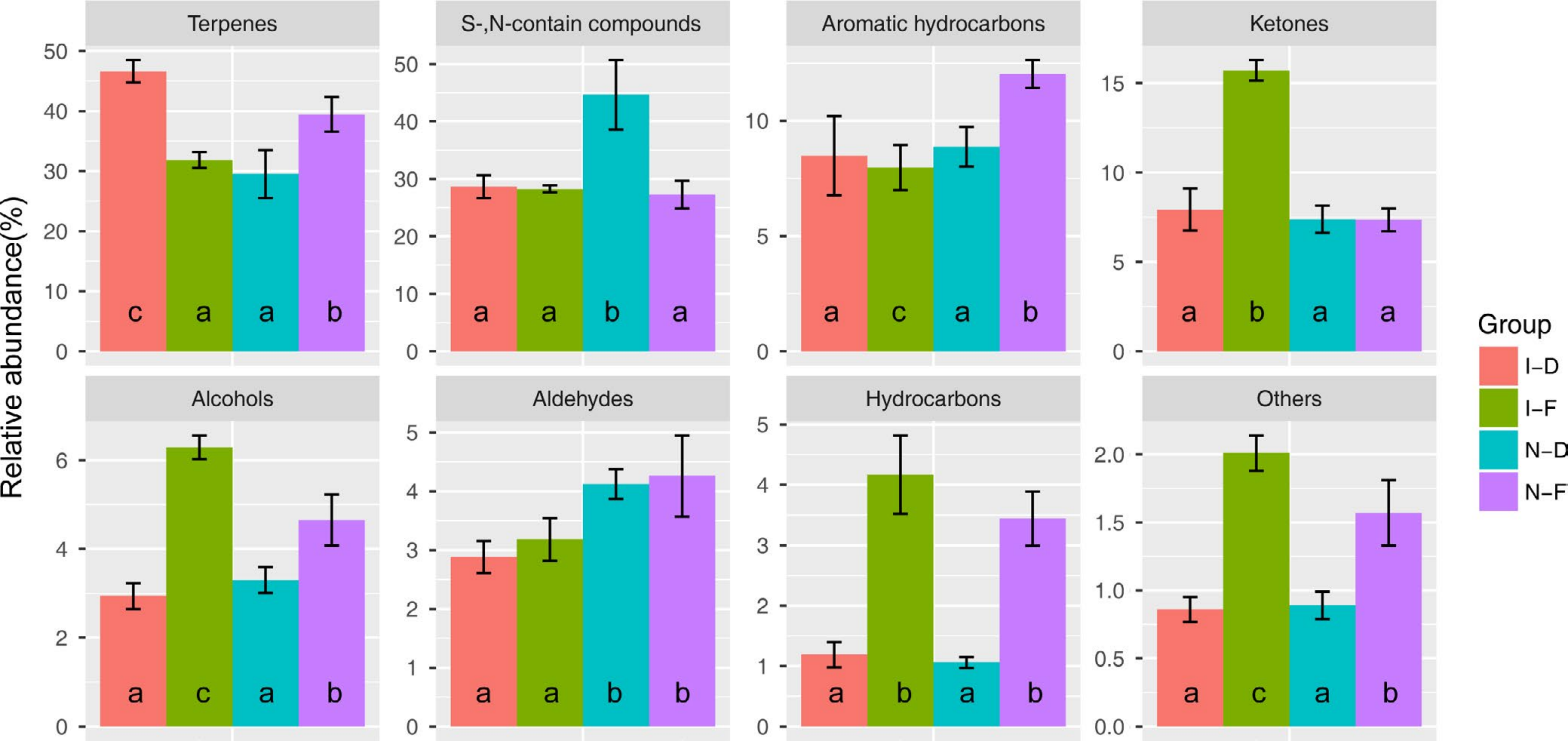
Proportion of the different chemical families of volatile compounds in jerky with different fermentation and cooking procedure (%)

We tentatively identified 73 volatile compounds, including terpenes, aromatic hydrocarbons, alcohols, aldehydes, ketones, esters, sulfur‐ and nitrogen‐containing compounds, and hydrocarbons (Figure 1). Both fermentation and cooking had notable effects on the generation of volatile compounds. Raw meat typically has little aroma (Lorenzo et al., 2014; Mottram, 1998a). Our analysis of beef jerky resulted in different volatile compound profiles for each treatment group. Figure 1 shows the relative percentages of the chemical groups found in the jerky treatments.

Volatile compounds can attain a balance during jerky processing, through thermal processing and biochemical pathways, and this balance is then lost by continuous evaporation or decomposition. The lowest number of volatile compounds (51) was detected in the I‐D group and the greatest in the IF group (62). The most abundant compounds extracted were terpenes, which originated from added spices, and sufur‐ and nitrogen‐containing compounds (S‐ and N‐containing compounds) derived from Maillard reactions. The highest proportions of terpenes (46.63%) and S‐ and N‐containing compounds (44.62%) were observed in the I‐D and N‐D groups, respectively. Aromatic hydrocarbons, ketones, and alcohols constituted 20%–30% of all volatile compounds, whereas the remaining chemical groups, hydrocarbons, aldehydes, esters, and others, accounted for only 5%–10%. A different chemical family profile was observed for each group, demonstrating the influence of fermentation conditions and cooking procedures on the volatile compounds formed during jerky production.

Most aldehydes were significantly affected by the postcooking procedures (*p* < .001) as shown in Table 3. The concentration of aldehyde produced by frying was more than drying, possibly linked to the oil in the frying process (Domínguez, Gómez, Fonseca, & Lorenzo, 2014). Generally, most aldehydes derived from lipid oxidation (i.e., hexanal, octanal, nonanal, 2‐decenal, (E)‐, tetradecanal, and hexadecanal) were increased by both fermentation and postcooking procedures. The main aldehyde of hexanal associated with off‐flavor has higher concentrations in fried jerky than in dried jerky (*p* < .001), which originated from the peroxidation of unsaturated fatty acids (Olivares, Navarro, & Flores, 2011). In the N‐F group, the concentration of 3‐methylbutanal was the highest (*p* < .01), whereas succindialdehyde was the lowest (*p* < .05). Branched aldehydes such as 3‐methylbutanal are formed mainly by Strecker degradation of leucine (Huang & Ho, 2001). Most aldehydes arising from fatty acid oxidation have low odor threshold values and may play an important role in beef jerky flavor (Mottram, 1998b; del Pulgar, Roldán, & Ruiz‐Carrascal, 2013). However, high levels of some aldehydes, including hexanal, can lead to rancidity (Ramirez & Cava, 2007). Changes in the types and concentrations of volatile compounds in jerky can result from variations in fermentation and postprocessing, due to the effects of the starter during fermenting and moderately high‐temperature frying. The most abundant aldehydes were hexanal and 3‐methylbutanal, which respectively come from amino acids degradation and unsaturated fatty acids oxidation.

**Table 3:** Selected volatile compounds (μg/kg) in jerky with fermentation (F) and postcooking procedure (C) (*n* = 4)

	LRI^1^	ID^2^	I‐F	N‐F	I‐D	N‐D	SEM	F	C	F × C
Aldehydes										
Butanal, 3‐methyl‐	643	a	44.31^c^	21.04^b^	7.82^a^	7.79^a^	4.94	**	***	**
Succindialdehyde	796	b	8.63^a^	44.94^b^	40.38^b^	41.08^b^	4.92	**	*	**
Hexanal	806	a	154.12^b^	155.61^b^	5.37^a^	5.38^a^	25.56	ns	***	ns
Octanal	1,005	a	15.65b	4.62^a^	nd	nd	2.65	**	—	—
Nonanal	1,104	a	4.86^ab^	6.44^b^	nd	3.59 ^a^	0.52	ns	*	—
Benzaldehyde, 4‐methoxy‐	1,171	c	18.14^c^	7.73^b^	7.32^b^	2.37^a^	1.87	***	***	*
2‐Decenal, (E)‐	1,212	b	nd	nd	18.51^a^	17.27^a^	1.07	ns	—	—
Tetradecanal	1,601	b	44.41^b^	nd	5.30^a^	32.58^b^	6.36	**	**	—
Hexadecanal	1,800	b	7.72^a^	nd	55.25^c^	30.39^b^	8.11	**	***	—
Total			296.24^c^	240.38^b^	139.96^a^	139.78^a^	21.49	ns	***	ns
Ketones										
Acetone	455	b	110.74	nd	nd	nd	—	—	—	—
Acetoin	717	a	1,124.61^b^	233.76^a^	247.15^a^	114.70^a^	156.23	***	***	***
5‐Hepten‐2‐one, 6‐methyl‐	938	c	52.96^a^	73.78^b^	80.34^b^	73.40^b^	4.22	ns	*	*
5‐Methyl‐4‐octanone	988	c	60.77^b^	57.29^ab^	56.50^ab^	51.35^a^	1.55	ns	ns	ns
2,3‐Octanedione	1,088	a	119.54^c^	46.58^b^	nd	9.75^a^	18	***	***	—
Total			1,469.10^c^	411.40^b^	384.00^b^	249.21^a^	148.00	***	***	***
Alcohols										
1‐Butanol	662	b	339.80^b^	67.44^a^	50.02^a^	45.45^a^	35.68	***	***	***
1‐Butanol, 3‐methyl‐	697	b	nd	66.99^b^	46.65^a^	39.44^a^	5.35	ns	*	—
2,3‐Butanediol	743	a	60.75^b^	53.69^b^	nd	8.73^a^	9.42	ns	**	—
1‐Octen‐3‐ol	969	a	72.07^c^	37.86^b^	6.88^a^	7.42^a^	8.83	***	***	***
2‐Octanol	979	b	16.73	nd	nd	nd	—	—	—	—
1‐Hexanol, 2‐ethyl‐	995	b	30.80^b^	9.19^a^	9.72^a^	8.71^a^	3.1	***	***	***
2‐Octen‐1‐ol, (E)‐	1,067	c	12.26^b^	nd	nd	7.11^a^	3.17	*	nd	nd
Phenylethyl Alcohol	1,136	b	55.62^d^	17.59^b^	30.25^c^	7.79^a^	6.38	***	***	**
Total			588.19^c^	259.88^b^	143.52^a^	111.47^a^	57.17	***	***	***
Terpenes and related compounds										
3‐Carene	948	c	34.00^b^	8.90^a^	nd	7.33^a^	4.36	***	ns	—
D‐Limonene	1,018	a	327.32^b^	189.69^ab^	44.93^a^	52.78^a^	39.29	ns	**	ns
Eucalyptol	1,059	c	nd	17.60^a^	79.11^b^	25.00^a^	12.76	*	ns	—
1,6‐Octadien‐3‐ol, 3,7‐dimethyl‐	1,082	c	163.33^b^	113.95^b^	94.17^b^	51.30^a^	47.54	**	**	—
2‐Ethyl‐3‐methoxy‐2‐cyclopentenone	1,106	c	nd	nd	41.31^b^	12.09^a^	6.26	***	—	—
L‐Fenchone	1,121	c	39.23^c^	24.98^b^	nd	10.49^a^	4.5	**	**	—
Isopinocarveol	1,131	c	10.20^b^	5.45^a^	nd	nd	1.08	**	—	—
Terpinen‐4‐ol	1,137	b	28.90^c^	14.04^ab^	21.76^bc^	9.16^a^	2.62	**	ns	ns
Isoborneol	1,138	c	105.36^c^	81.07^bc^	64.27^ab^	39.62^a^	9.02	*	**	ns
p‐Menth‐8‐en‐1‐ol, stereoisomer	1,158	c	4.69	nd	nd	nd	—	—	—	—
Estragole	1,172	b	151.31^d^	60.71^b^	105.89^c^	29.92^a^	14.94	***	**	ns
Anethole	1,190	b	1,626.57^c^	1,421.65^bc^	1,142.45^b^	597.65^a^	137.7	*	**	ns
β‐ylangene	1,216	c	16.25^bc^	13.31^b^	19.67^c^	7.73^a^	1.44	**	ns	*
Copaene	1,221	c	38.98^c^	16.32^b^	39.50^c^	4.84^a^	4.82	**	***	***
Geranyl vinyl ether	1,250	c	13.88^b^	8.86^a^	8.85^a^	5.19^a^	1.1	**	**	ns
1,5,5‐Trimethyl‐6‐methylene‐cyclohexene	1,338	c	16.62^a^	9.31^a^	nd	nd	1.97	ns	—	—
Aromandendrene	1,386	c	29.84^b^	nd	32.19^b^	7.03^a^	4.39	***	ns	—
Cyclohexene, 3‐(1,5‐dimethyl‐4‐hexenyl)‐6‐methylene‐, [S‐(R*,S*)]‐	1,446	c	51.38^c^	26.21^b^	47.14^c^	11.79^a^	5.67	***	**	*
β‐curcumene	1,480	c	43.77^a^	29.29^a^	97.59^b^	30.31^a^	10.74	**	**	**
Caryophyllene	1,494	b	246.72^c^	121.24^b^	270.29^c^	48.73^a^	29.53	***	ns	*
β‐Bisabolene	1,500	b	52.67^b^	nd	69.80^c^	20.89^a^	7.63	***	ns	‐
Benzene, 1‐(1,5‐dimethyl‐4‐hexenyl)‐4‐methyl‐	1,524	c	108.68^c^	49.13^b^	89.41^c^	26.19^a^	10.84	***	*	ns
Total			2,976.99^c^	2,211.69^b^	2,268.34^b^	991.31^a^	217.90	***	***	*
Hydrocarbons										
*n*‐Hexane	618	b	48.57	63.91	nd	nd	5.14	ns	—	—
Decane	1,015	b	14.57^a^	12.17^a^	13.52^a^	nd	1.15	ns	ns	—
Undecane	1,115	b	34.04^b^	36.18^b^	37.98^b^	4.80a	4.51	*	*	*
Dodecane	1,214	b	132.29	nd	nd	nd	—	—	—	—
Tridecane	1,313	b	159.82^c^	57.35^b^	7.07^a^	6.61^a^	21.83	**	***	**
Tetradecane	1,413	b	nd	nd	24.43^a^	22.99^a^	2.78	ns	nd	nd
Total			389.30^c^	192.60^b^	58.57^a^	35.84^a^	43.06	***	***	**
Aromatic hydrocarbons										
Styrene	883	b	457.08^b^	462.04^b^	296.41^a^	227.67^a^	36.4	ns	**	ns
Ethylbenzene	893	b	28.28^ab^	37.72^b^	19.50^a^	16.12^a^	3.12	ns	**	ns
p‐Xylene	904	b	123.68^b^	30.09^a^	9.43^a^	11.53^a^	17.41	*	**	*
Benzene, 1,3‐dimethyl‐	906	b	35.88^ab^	61.25^b^	41.49^ab^	25.17^a^	5.22	ns	ns	*
o‐Xylene	907	b	25.13^a^	11.58^a^	19.07^a^	14.67^a^	2.59	*	ns	*
Mesitylene	1,020	b	nd	3.51	nd	nd	—	—	—	—
o‐Cymene	1,041	b	34.74^b^	4.58^a^	nd	nd	7.08	**	—	—
p‐Cymene	1,042	b	nd	5.51	nd	nd	—	—	—	—
Naphthalene	1,231	b	nd	47.07	nd	nd	—	—	—	—
Benzocycloheptatriene	1,251	c	nd	11.13^b^	9.03^b^	1.96^a^	1.44	**	***	ns
Naphthalene, 1‐methyl‐	1,345	c	10.95^b^	nd	16.72^c^	3.25^a^	1.77	ns	***	**
Total			742.90^b^	674.50^b^	416.54^a^	299.76^a^	57.16	*	***	ns
Sulfur and nitrogen compounds										
Disulfide, dimethyl	722	b	nd	11.46^b^	nd	3.53^a^	2.29	—	**	—
Trimethylpyrazine	1,008	b	343.21^b^	404.30^c^	189.84^a^	164.55^a^	33.76	ns	***	*
Ethanone, 1‐(1H‐pyrrol‐2‐yl)‐	1,035	c	8.63	nd	8.82	nd	0.23	—	ns	—
3‐Methyl‐2‐thiophenecarboxaldehyde	1,072	c	10.9	nd	nd	nd	—	—	—	—
Pyrazine, 2‐ethyl‐3,5‐dimethyl‐	1,107	c	19.87^ab^	31.67^b^	29.30^ab^	17.26^a^	2.43	ns	ns	*
Pyrazine, tetramethyl‐	1,121	b	2,225.32^b^	1,071.92^a^	1,171.23^a^	1,345.13^a^	162.96	*	ns	**
4H‐Pyran‐4‐one, 2‐ethyl‐3‐hydroxy‐	1,163	c	29.24	nd	nd	nd	—	—	—	—
1,2,4‐Triazolo[4,3‐b]pyridazine, 8‐methyl‐	1,194	c	nd	nd	nd	8.58	—	—	—	—
Benzothiazole	1,208	b	5.66^a^	6.76^ab^	nd	8.51^b^	0.56	ns	*	—
Total			2,641.17^b^	1,534.94^a^	1,398.88^a^	1,537.51^a^	163.67	*	**	**
Others										
Benzene, 1,4‐dichloro‐	1,038	c	20.35^c^	15.06^b^	5.79^a^	5.89^a^	1.87	ns	***	ns
Dichloroacetic acid 2‐methylpropyl ester	1,066	c	20.86^c^	10.11^b^	3.22^a^	nd	2.93	***	***	—
Trichloroacetic acid 2‐methylpropyl ester	1,108	c	111.73^b^	48.30^a^	nd	nd	14.68	**	—	—
γ‐Chlorobutyrophenone	1,453	c	34.10^c^	25.06^b^	32.88^c^	12.66^a^	2.96	***	*	*
Total			187.26^c^	88.41^b^	41.89^a^	30.27^a^	18.80	***	***	***

Note

^a–d^Means within the same row with different superscript upper case letters are different (*p* < .05).

Abbreviations: nd, not detected; ns, not significant; *SEM*, standard error of the mean.

^1^ Linear retention index in the TR‐5 column.

^2^ Peak identification: a, comparison of spectra and retention time with commercial standards; b, identified by spectra comparison using the Wiley library and LRI comparison with literature; and c, tentative identification by mass spectrum.

* *p* < .05.

** *p* < .01.

*** *p* < .001.

Ketones in jerky were significantly influenced by the fermentation and cooking procedures (*p* < .001). As shown in Table 3, five ketones were identified, most of which had low concentrations, except for acetoin. These ketones were probably generated via both biochemical pathways and thermal processing (Lorenzo et al., 2014). Acetoin is an important aromatic substance that originates from Maillard reactions (Mottram, 1998b). The I‐F group contained higher acetoin levels than those of all other groups (*p* < .001), likely because lactic acid bacteria in starter cultures can produce acetoin via pyruvate metabolism (Montanari et al., 2016). Acetoin can form at low temperatures (e.g., 80°C) and react with ammonia to form heterocyclic aroma compounds (Liu, Liu, He, Song, & Chen, 2015; Xi, Huang, & Ho, 1998). Levels of 2,3‐octanedione were highest in the N‐F group (*p* < .001) due to pyruvate metabolism in the starter culture (Montanari et al., 2016).

Alcohols were significantly influenced by the addition of starter culture and the postcooking procedures (Table 3). Overall, the I‐F group produced the highest content of volatile compounds, of which 1‐butanol was the most abundant (*p* < .001). 1‐butanol (the mellow note) formation can be the result of pyruvate metabolism, which is important for flavor; they can also mask rancid odors. Montanari et al. (2016) reported that 1‐butanol was the aroma compound most associated with starter culture. However, most saturated alcohols have a higher threshold value and contribute little to jerky flavor (Marušić et al., 2014). In contrast, some unsaturated alcohols such as 1‐octen‐3‐ol have a low threshold value; 1‐octen‐3‐ol was presented in relatively high proportions in the I‐F group (~72 μg/kg) and has been reported to provide aromatics similar to those of mushrooms (Carrapiso & Garcia, 2004). This compound is also reported to be abundant in fried Chinese‐style jerky, in which it is likely formed by lipid oxidation (Yang et al., 2017). Heat treatment and culture addition play an effective role in fatty acid decomposition. Levels of 2,3‐butanediol (butter) were significantly higher in fried jerky than in dried jerky (*p* < .01), and 2,3‐butanediol is produced by the reduction of methyl ketones from fatty acid α‐oxidation (Marco, Navarro, & Flores, 2006). These volatiles can be derived from microbial activities involving carbohydrate fermentation and are often detected in fermented jerky (Lorenzo et al., 2012; Marco, Navarro, & Flores, 2008; Summo, Caponio, Pasqualone, & Gomes, 2011).

Terpenes were the highest in proportion among chemical groups in all jerky groups (Table 3). Due to the spice treatment on the jerky surface during processing, some terpenes were generated from added pepper and laurel (Hinrichsen & Pedersen, 1995). The overall production of terpenes was influenced (*p* < .001) by culture addition and cooking procedure (*p* < .001). Terpene concentrations were lowest in the N‐D group, suggesting that they were lost via evaporation during the drying process. In the N‐D group, the compounds with the highest concentrations included anethole, d‐limonene, caryophyllene, estragole, and isoborneol. In particular, d‐limonene and anethole levels were significantly higher in the I‐F group than in all other groups (*p* < .001).

Six alkanes were detected in the jerky samples produced in this study (Table 3). Overall, the production of hydrocarbons was influenced by culture addition and cooking procedures (*p* < .001). Alkanes such as undecane and tridecane were detected at high levels. Undecane levels were lower in the N‐D group than in all other groups (*p* < .05). However, tridecane levels were higher in fried jerky than in dried jerky (*p* < .001). Alkanes with fewer than 10 carbon atoms are derived mainly from fatty acid oxidation (Mottram, 1998a; Wettasinghe, Vasanthan, Temelli, & Swallow, 2001), and the high temperatures attained during cooking also generate a wide range of alkanes (Min & Ahn, 2005). Alkanes with longer chains typically accumulate in animal fat deposits, probably as a result of feed type (Meynier, Novelli, Chizzolini, Zanardib, & Gandemera, 1999). During frying, high temperatures can also stimulate lipid peroxidation.

As shown in Table 3, fermentation and cooking procedures significantly affected the abundance of aromatic hydrocarbons. Styrene was the most abundant aromatic hydrocarbon among all groups, with higher levels in fried jerky than in dried jerky (*p* < .01). Aromatic hydrocarbons such as styrene, ethylbenzene, and 1,3‐dimethylbenzene contribute little to the flavor of jerky because of their high threshold values, but improve overall flavor of jerky (Ramirez & Cava, 2007). These substances (e.g., xylene and styrene) are presented in plants consumed by animals (Meynier et al., 1999).

Many miscellaneous compounds were also detected, including S‐ and N‐containing compounds (Table 3). These have very low odor thresholds and may determine jerky flavor (Mottram, 1998b). Heterocyclic compounds such as pyrazine and thiazole are formed via Maillard reactions (Chen, Liu, & Chen, 2002). S‐containing compounds form during the degradation of amino acids (methionine) and thiamine (Roldán, Ruiz, Pulgar, Pérez‐Palacios, & Antequera, 2015). We detected higher levels of tetramethylpyrazine in fried jerky than in dried jerky (*p* < .001), indicating that levels of N‐containing heterocyclic compounds (pyrazines and pyridines) increased at high temperatures (>100°C) after 30 min of heating, during frying treatment (Liu et al., 2015). Pyrazines are a major class of volatiles in grilled meat (Mottram, 1998b). Thus, cooking temperatures can increase the already high abundance of volatile compounds derived from Maillard reactions.

## 4 CONCLUSIONS

The results of this study provided evidence that fermentation and postcooking procedures influenced various characteristics of jerky. The *a*_w_, L*, and a* values of beef jerky were affected by cooking treatment. Hardness, chewiness, and resilience were lower after drying than after frying. Fried samples prepared with starter culture contained the highest levels of volatile compounds among all groups. The most abundant volatile compound groups were terpenes and S‐containing compounds, followed by aromatic hydrocarbons, ketones, and alcohols. Overall, color and tenderness were best in the IF group, which had the highest content of volatile compounds, ultimately resulting in the formation of a stronger flavor.

## CONFLICT OF INTEREST

The authors declare that they have no conflicts of interest.

## ETHICAL APPROVAL

This study does not involve any human or animal testing.
